# Development of a Food Literacy Assessment Tool for Healthy, Joyful, and Sustainable Diet in South Korea

**DOI:** 10.3390/nu14071507

**Published:** 2022-04-04

**Authors:** Hyelim Yoo, Eunbin Jo, Hyeongyeong Lee, Sohyun Park

**Affiliations:** 1 Department of Food Science and Nutrition, Hallym University, Chuncheon 24252, Korea; yhyelim@hallym.ac.kr (H.Y.); m20514@hallym.ac.kr (E.J.); gusrud0303@hallym.ac.kr (H.L.); 2 The Korean Institute of Nutrition, Hallym University, Chuncheon 24252, Korea

**Keywords:** food literacy, definition, validity, questionnaire, eating behavior, sustainable eating habits

## Abstract

Background: Food literacy (FL) is important as the ability to consider the unique aspects of food in our lives, society, and environment. The main objectives of this study were as follows: (1) to revisit the definition of FL, considering the cultural, relational, and ecological aspects that were often neglected in previous research, and (2) to develop a measurement tool for adults. Methods: Expert workshops, the Delphi survey, the test–retest survey, and one-on-one interviews were conducted. The content validity ratio was calculated from the Delphi survey. The correlation coefficient of each item was measured twice, and the Cronbach’s alpha was calculated. Results: This study proposed a new definition of FL, including future-oriented values, and suggested three main domains with 33 items: (1) 14 questions in nutrition and safety FL (Cronbach’s α = 0.877, average correlation coefficient = 0.70), (2) 8 questions in cultural and relational FL (Cronbach’s α = 0.705, average correlation coefficient = 0.71), and (3) 11 questions in socio-ecological FL (Cronbach’s α = 0.737, average correlation coefficient = 0.61). Conclusions: This newly developed questionnaire should be tested in different populations; however, this questionnaire can be a basis for measuring and improving FL for healthy, joyful, and sustainable diets for adults.

## 1. Introduction

The prevalence of chronic diseases such as obesity, high blood pressure, and diabetes is steadily increasing, and poor eating habits such as skipping breakfast, eating out, and reduced fruit and vegetable intakes are also on the rise [1]. The effects of dietary intake on health are significant [2] and switching to a healthier diet can potentially reduce chronic conditions such as obesity, cardiovascular disease, and diabetes [3].

Recently, there has been increasing interest in sustainable diets, as well as individual nutrition choices for health [4,5]. Sustainable diets aim to ensure that what we eat has a minimal impact on the environment while contributing positively to the lives of future generations [6,7]. Since environmental impacts vary depending on the type of diet we follow, the ability to think about how our diet can affect our health and the environment is important. In order to improve our quality of life and to follow an environmentally sustainable diet, we need to have the ability to understand and choose the proper foods [8]. However, so-called “food literacy” has only recently been actively discussed.

Food literacy is originally derived from health literacy (HL), which is defined as the “cognitive and social skills that determine the ability to access, understand, and use information to promote and maintain individual health”, suggested by Nutbeam [9,10]. Previous literature defining the key domains of food literacy, therefore, focused on the three main domains adapted from HL: namely, the functional, interactive, and critical domains [8]. There are several versions of food literacy definitions in the literature. In general, the functional domain is the ability to acquire, understand, and use food-related information. The interactive domain is the ability to obtain and provide information regarding food through various forms of communication and the ability to apply it to changing environments. Lastly, the critical domain is the ability to critically evaluate and act on food-related information [9,10,11].

### The Review of Food Literacy

Food literacy was mentioned by Vidgen [12] and Cullen [13], who emphasized that individuals tend to function better if they develop positive relationships with food in complex food systems, as well as food-related knowledge, skills, and behaviors for healthy eating habits, and make informed, health-related decisions. However, it is necessary to look at food literacy in a broader sense, so that food can be selected sustainably by considering its cultural, community, societal, and environmental values [8].

Food literacy measurement tools are being actively developed in many countries, but measurement tools that include all three main domains are scarce. Among the questionnaires containing all three domains, most of them focus on measuring only one domain, mostly the functional aspect of food literacy [8,14,15,16,17], and none of them include all the core domains in a broad sense, particularly for sustainable eating.

For example, Stjernqvist divided the three main domains of food literacy into five “abilities”. However, in the “To Want” ability, to participate and act with responsibility and will in relation to the environment, there were too few questions related to environmentally conscious packaging, recycling, and ecologically sustainable food. In the “To Care” ability, which considered the individual, the surrounding communities, and social factors, there were also too few questions regarding inequality or sustainable “win-win” food-related practices, such as Fair Trade [18].

Park et al. (2020), by contrast, divided their questionnaire into three domains based on Nutbeam’s HL model and on areas of the food system level that take societal and environmental sustainability into consideration. However, the questionnaire lacked items regarding the relationship between the climate crisis and the food crisis, inequality and food justice, and the pursuit of pleasure and meaning through food [19].

Furthermore, it is important to consider the unique aspects of food in our lives, societies, and environments, which cannot be covered by the main domains adapted from HL. For example, the cultural and ecological aspects of food choices and food justice for all participants in food systems are becoming more crucial in the current society. However, matching these unique aspects of food to predefined health literacy domains is not sufficient to fully cover the complex concept of food literacy.

Thus, the main objectives of this study were as follows. First, we revisited the concept of food literacy, considering the cultural, relational, and ecological aspects, as well as individual ability to eat nutritiously and healthy. This included future-oriented core values that have social (eating in equality) and environmental issues (food waste, packaging waste, and meat consumption). Second, we developed a food literacy questionnaire with proven validity that can be used for future dietary education and policy development and evaluation.

## 2. Materials and Methods

### 2.1. General Overview of the Study Process

**Figure 1:**
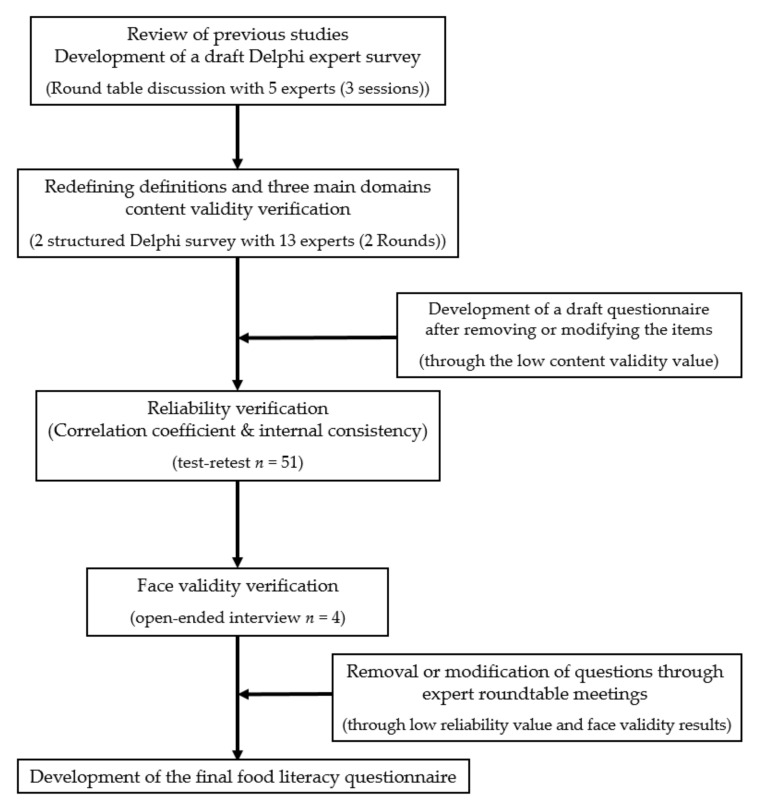
Flow chart for establishing the concept of food literacy and the questionnaire development.

Figure 1 outlines the process for redefining the concept and the main domains of food literacy and developing a measurement tool. First, definitions for the main domains of food literacy were determined by reviewing previous studies and expert workshops, which led to the development of a draft Delphi expert survey. Next, the definition and three main domains of food literacy were redefined by conducting the Delphi survey of experts, and the content validity of the questionnaire was verified. Based on the results of the Delphi survey, a reliability test was conducted by analyzing the correlation coefficient through the test–retest method and internal consistency through Cronbach’s alpha for general adults after developing the draft questionnaire. Next, one-on-one interviews were conducted with general adults to verify face validity and the understanding of the questionnaire items. The final questionnaire on food literacy based on the reliability and validity results was then completed during an expert roundtable meeting. All the participants provided written consents, and the study protocol was approved by the Institutional Review Board of Hallym University (HIRB-2021-009).

### 2.2. Expert Workshops and the Expert Delphi Surveys

The expert panel consisted of individuals who have worked in the fields of food and nutrition, food policy, and food inequality for more than 10 years. Some experts were selected from the list of the policy advisory group for the government of Seoul. A total of three expert workshops were conducted, during which the definition and three main domains of food literacy were specified, a set of questions measuring food literacy was developed, and a panel of experts was selected to create a draft Delphi expert survey.

The content validity ratio (CVR) was calculated to verify the validity of the Delphi survey:

$$\frac{Ne-N}{N}$$

where *Ne* is the number of panel experts who responded that the item was important, and *N* is the total number of expert panelists who responded. The CVR calculates a minimum value according to the number of panels, and the item content is judged as valid if it meets or exceeds the minimum value [20].

The Delphi survey panel was active in the areas of production, manufacturing/processing/packaging, consumption, distribution/sales, and environment in the food system. A total of 15 experts were selected for the panel, which comprised 10 practitioners/policy experts and 5 researchers from academia. The Delphi survey was conducted twice. The first survey focused on opinions surrounding the definition of the three main domains of food literacy, opinions on the necessity and revision of food literacy questionnaires, and open-ended questions as free opinions for each domain. The second survey focused on the specific items for measuring food literacy based on the revised definitions and three main domains and on calculating the CVR of the suggested items.

### 2.3. Reliability and Face Validity

In June 2021, the survey was conducted using the draft questionnaire at one-week intervals to obtain the correlation coefficient of the test–retest. A convenient sample of 51 adults were recruited to include both men and women from their 20s to 50s. The online survey link with research explanations and consent forms for participation was sent to the participants. For the repeated survey, the phone numbers of the participants were collected, and a week after the first survey, another link was sent to the participants. Based on the survey responses, the correlation coefficient of each item’s repeated measurements was analyzed using STATA 17.0. The correlation coefficient was compared and analyzed using the responses of 45 out of the 51 subjects. Six were excluded, as their answers deviated significantly between the first and second surveys. The correlation was judged to be very high if the correlation coefficient was 0.9 or more, high if it was between 0.7 and 0.9, relatively high between 0.4 and 0.7, low between 0.2 and 0.4, and no correlation if it was less than 0.2 [21,22].

In order to examine the internal consistency of the questions, Cronbach’s α was calculated for the three main domains. The internal consistency was determined to be very reliable if the value was between 0.8 and 1.0, reliable between 0.6 and 0.79, moderately reliable between 0.4 and 0.59, moderate between 0.2 and 0.39, and unreliable if the value was 0.2 or less [21,23].

To calculate the face validity and the understanding of the food literacy questionnaire, one-on-one interviews were conducted with four individuals. The subjects were interviewed by looking for convenience samples in their 20s and 40s according to whether they participated in the survey and their experiences related to food literacy.

## 3. Results

### 3.1. Validity and Reliability Assessment

#### 3.1.1. Delphi Survey: Modified Food Literacy Definition and Components of the Main Domains

The Delphi survey was conducted twice in total, and 13 out of the 15 experts invited responded. When discussing the definition of food literacy, the Delphi experts expressed opinions such as “I think health maintenance serves a more comprehensive purpose than health promotion”, “We should mention cultural value”, and “We should ensure the definition considers the value of the community, agriculture, and the environment”.

Based on the expert opinions from the first Delphi survey, modified definitions of food literacy were agreed upon as follows. The initially suggested definition to the panel—that is, “the ability to select, prepare, and cook food to promote good health as well as food-related competencies that take into account the value of the community, agriculture, and the environment”—was modified to “the ability to select, prepare, and cook food to promote a healthy lifestyle, as well as food-related competencies that understand the cultural value of food, and which take into account the value of food to the community, agriculture, and the environment”.

The modified definition and components of the three main domains based on expert opinions from the first Delphi survey are shown in Table 1. In short, the functional literacy was changed to “nutrition and safety”, the interactive literacy was changed to “cultural and relational”, and the critical literary was changed to “socio-ecological” domains.

#### 3.1.2. Content Validity Ratio (CVR) Results from the Delphi Survey

The CVR value was analyzed using values of 1 (not necessary), 2 (somewhat necessary), and 3 (absolutely necessary) to agree on the necessity of questionnaire items by each domain of food literacy. The first and second Delphi surveys were judged to be appropriate when the CVR exceeded 0.54 [20]. After the first Delphi survey, the second Delphi survey was conducted after modifying or removing any items with low CVR values. The content validity results of the 34 questions after the second Delphi survey are shown in Table 2. Based on the results of the content validity and the opinions of experts on the predicate of questions, a draft food literacy questionnaire was developed.

#### 3.1.3. Reliability (Test–Retest Reliability and Internal Consistency)

Table 3 shows the general characteristics of the subjects who participated in the test–retest survey. A total of 51 subjects (32 women and 19 men) participated in the repeated survey one week apart. When looking at the deviation between the two responses to determine response errors, six subjects (based on outliers) who had values of 1.5 times or greater than the quartile range were considered to have given insincere responses and were excluded. Therefore, the correlation coefficient and internal consistency (Cronbach’s α) of the scores of 45 subjects in total were analyzed. The correlation coefficients were between 0.4 and 0.9, except for Question Item #27: “I think rural farmers are important for a sustainable society”.

The internal consistency within each domain showed all a good level of reliability. Specifically, for the nutrition and safety domain, the overall internal consistency, measured with Cronbach’s α, for the second survey was 0.8772, showing a good level of internal consistency. In the cultural and relational domain, the overall internal consistency for the second survey was 0.7048, showing a good level of internal consistency. In the socio-ecological domain, the Cronbach’s alpha results showed a good reliability level of 0.7373 (Table 2). There were no items that significantly increased the internal consistency when removed in all these domains.

#### 3.1.4. Face Validity

The interviewees were housewives in their 40s who did not participate in the survey but believed that they had a lot of experience related to food literacy as defined by us. The other interviewees were selected from adults in their 20s who had participated in the survey. Their opinions on their understanding of the questionnaire items analyzed through the open-ended interviews was that it was necessary to explain the wording of some questionnaire items (e.g., information related to food safety). In the socio-ecological domain, there were several professional-level questions that could only be understood fully if the respondents had a high level of food literacy and had an interest in the usual. Therefore, revising some questionnaire items was thought to be necessary.

#### 3.1.5. Development of the Final Questionnaire

Questionnaire items were modified or removed depending on their reliability and validity. The questionnaire item, “I know that food selection affects health” was excluded, as it was the only one of the 34 questions to have a low correlation coefficient between test and retest. It was also thought that the questionnaire items “I know about the various food groups that make up nutritionally balanced meals” and “I try to eat a variety of food groups such as cereals, fish, vegetables, fruits, and dairy” would be acceptable substitutes.

Therefore, the final version of the food literacy questionnaire consisted of 33 questions: 14 in the nutrition and safety domain, 8 in the cultural and relational domain, and 11 in the socio-ecological domain (Appendix A, Table A1).

## 4. Discussion

Since food literacy is contextual [12], various contextual factors must be considered, along with basic abilities and behaviors related to food. Although studies related to food literacy are actively being discussed, there are relatively little food literacy studies that consider future-oriented core values such as social issues (e.g., inequality and coexistence related to food) and ecological–environmental issues (e.g., the climate crisis related to food, food waste, packaging waste, etc.).

This study established a new definition of food literacy that included future-oriented values and suggested three main domains, which can allow people to make the correct judgments and choices for a healthier, happier, and more sustainable diet. We considered the cultural, social, ecological, and environmentally unique aspects of food that have not been covered in previous studies, along with the existing food literacy concept. Therefore, various emotional and relational components through food and components that consider sustainable dietary and coprosperity, such as climate crisis and inequality, were included.

There have been no questionnaires that discussed a food literacy management tool in South Korea, except for the recently developed Korean questionnaire by Park et al. (2020). Other food literacy questionnaires have been developed by researchers in various countries, but the questionnaire items tend to focus only on one aspect of food literacy rather than including various aspects of food literacy.

For example, the existing food literacy measurement tools were mostly developed with functional literacy, such as food and nutrition-related knowledge; understanding; and questions to plan, manage, prepare, and select food. There were few items dealing with the cultural and relational domain, which maintains a good relationship with food and shares with people, and the socio–ecological domain, which thinks about food sustainability and the environment. Especially, there were no items about food values, the climate crisis, greenhouse gas reduction, food justice, or inequality. Therefore, we developed a food literacy questionnaire regarding the understanding of food in a wide range of contexts that are related to core values that are rarely included in the current nutrition education or dietary intervention studies.

In spite of the unique contribution of this study, there are a few limitations that need to be addressed. First, the time intervals of the test and retest were somewhat short for accurately verifying the reliability. In addition, the sample size was small, meaning that the small number of subjects that had large deviations between the two tests significantly influenced the overall values. Further, surveys with larger sample sizes will need to be conducted, as it was difficult to accurately determine whether the deviations in their answers were because of untruthful responses or whether they misunderstood some questions. Additionally, due to time constraints, only a few interviews were conducted regarding the verification of face validity.

Content validity and face validity were verified, but an in-depth validity test such as criterion validity was not performed. By collecting more data, we will be able to establish the additional validity of indicators by employing analyses such as structural equation modeling.

Despite its limitations, this study not only attempted to measure individual food selection and cooking ability but also sustainable food selection capabilities in areas such as food justice and the current climate crisis. The developed questionnaires will be used to identify what kind of education is needed for a sustainable and healthy life and the target populations and to confirm the effectiveness of the educational programs. In addition, it can be utilized for food policy evaluation if a larger scale of a citywide surveillance system is in place. Additionally, it is important to keep in mind that all these processes should be implemented carefully to not victimize vulnerable populations. It is hoped that this research can be used for education and research purposes in the future and that it can be used to improve food literacy for everyone.

## 5. Conclusions

Food literacy can assist in promoting and maintaining an individual’s health by providing the information to make healthy food choices in a rapidly changing dietary environment and can serve as the foundation for following a sustainable diet in the future [24,25,26].

Food literacy has been focused on individuals’ capability of making healthy food choices. However, food literacy must also be about the ability to choose food in a sustainable manner that considers the community, the wider society, and the environment, as well as the health of the individual.

We have proposed a new, broader definition of food literacy that allows people to make the right choices for a healthy, joyful, and sustainable diet. We presented three main areas consisting of the nutrition and safety domain, cultural and relational domain, and socio-ecological domain. Finally, a food literacy measurement tool consisting of 14 questions in the nutrition and safety domain, 8 questions in the cultural and relational domain, and 11 questions in the socio-ecological domain was developed.

In the future, based on this study, it can be used to test the level of food literacy and explore related factors with larger sample sizes with various social backgrounds. Additionally, this tool can be used to identify the vulnerable groups of food literacy and, further, to plan and implement educational programs for these groups. Our food literacy questionnaire can hopefully be a valuable tool in program planning, education, and policy evaluation for a healthy and joyful diet in a sustainable environment.

## Figures and Tables

**Table 1:** The definition and components of the three main domains were modified based on the expert roundtable discussion and Delphi surveys.

Domains	Nutrition and Safety Food Literacy (FL)	Cultural and Relational FL	Socio-Ecological FL
Definition	- The ability to acquire, understand, and use knowledge related to food and nutrition and information related to ingredients and cooking.	- Interest and understanding of food culture - The ability to contribute to the promotion of individual and community life and wellbeing through food, the pursuit of enjoyment and meaning through food, the gastronomic interest in food.	- The ability to understand and value various social and ecological consequences related to individual food choices. Interest and perception of inequality or “win-win” practices related to food.
Components	- Diet planning and management, understanding and purchasing ingredients, storage, cooking - Knowledge, self-efficacy, attitude, practice, and utilization related to food safety and nutrition - The ability to discern and utilize correct and incorrect information related to food and nutrition	- The ability to understand and value various food cultures including our traditional food culture - Sharing food-related information, enjoying delicious food, enjoying cooking, keeping interest in eating and sharing food with others, and having gratitude for food	- Interest in local food and seasonal ingredients, interest in buying directly from producers, urban and rural coexistence, food waste, food packaging material and recycling for the environment, interest and recognition of eco-friendly/animal welfare/fair trade food ingredients, recognition of the relationship between the climate crisis and food crisis. - Interest and recognition of food inequality.

**Table 2:** CVR values of the 34 questions after two Delphi surveys, the correlation coefficient of the 5-point scale, internal consistency of the test–retest, and the change value of Cronbach’s α in item removal (*n =* 45).

Main Domain	Questionnaire Items	Content Validity Ratio (CVR)	Correlation Coefficient	Cronbach’s α in Item Removal
Nutrition and Safety FL	Q1. I know about the various food groups that make up nutritionally balanced meals.	1	0.6067	0.8682
	Q2. I know that food selection affects health.	1	0.4588	0.8784
	Q3. I try to eat a variety of food groups such as cereals, fish, vegetables, fruits, and dairy.	1	0.6208	0.8804
	Q4. I make a list of items I need to buy before grocery shopping.	0.85	0.8181	0.8866
	Q5. I can understand the food labeling in packages of processed foods.	1	0.8108	0.8676
	Q6. When purchasing processed food, I check food information (ingredients, nutrition facts).	1	0.8051	0.8605
	Q7. I check the country of origin when purchasing food.	1	0.7357	0.8713
	Q8. I know how to separate and store ingredients that I cannot consume immediately.	1	0.8046	0.8597
	Q9. I can follow a simple recipe.	0.85	0.8269	0.8679
	Q10. I can prepare a meal without difficulty.	1	0.8464	0.8612
	Q11. I wash my hands thoroughly before cooking.	0.54	0.7134	0.8730
	Q12. I know how to store food in refrigerator, or room temperature that can affect freshness and safety.	1	0.6680	0.8676
	Q13. I check the cleanliness of restaurants when eating out.	0.85	0.7586	0.8733
	Q14. I can understand information related to food safety issues in the media.	1	0.6129	0.8603
	Q15. I can judge critically about the food advertisement content, especially the health claims.	1	0.4444	0.8631
	Total Cronbach’s α			0.8772
Cultural and relational FL	Q16. Cooking is enjoyable.	1	0.8467	0.6719
	Q17. When eating, I fully concentrate on eating.	0.69	0.6123	0.7102
	Q18. When eating, I savor various senses such as visual beauty, aroma, taste, and texture.	0.85	0.5623	0.6530
	Q19. I am grateful for the process that has allowed the food to come to the table.	0.85	0.7609	0.6289
	Q20. I like to eat or share food with my family, friends, and neighbors.	1	0.7869	0.6730
	Q21. I enjoy talking about food with people around me.	1	0.7436	0.6373
	Q22. I am interested in food from various cultures.	0.85	0.7356	0.7118
	Q23. Enjoying traditional food can help protect our cultural identity.	0.69	0.6079	0.7063
	Total Cronbach’s α			0.7048
Socio-ecological FL	Q24. I know why choosing seasonal food is good for the environment.	1	0.7178	0.7072
	Q25. I think food that is directly traded with producers is more reliable.	1	0.4556	0.7219
	Q26. I think choosing organic products is important for environmental conservation.	1	0.5629	0.7391
	Q27. I think rural farmers are important for a sustainable society.	0.69	0.3248	0.7093
	Q28. I am interested in urban agriculture (such as city gardening, weekend farming, etc.).	0.85	0.7937	0.7279
	Q29. It is important to consider animal welfare when purchasing meat and eggs.	1	0.5734	0.6928
	Q30. I know why it is better to choose fair-trade products.	1	0.7701	0.7262
	Q31. I believe that reducing meat and promoting vegetarianism helps slow climate change.	1	0.6420	0.6936
	Q32. I try to reduce food waste.	1	0.6379	0.7452
	Q33. I try to reduce food packaging waste (take-out drinks, delivery foods, etc.).	1	0.5528	0.6934
	Q34. I think everyone should have access to quality food regardless of economic circumstances.	1	0.7127	0.7360
	Total Cronbach’s α			0.7373

**Table 3:** Characteristics of the subjects who participated in the test–retest.

Subject	Total (*n*, %)	Men (*n*, %)	Women (*n*, %)
Total	51 (100.0)	19 (37.3)	32 (62.7)
Age			
20–39	32 (100.0)	11 (34.4)	21 (65.6)
40–59	19 (100.0)	8 (42.1)	11 (57.9)
Education level			
High school graduate	7 (100.0)	2 (28.6)	5 (71.4)
College student	7 (100.0)	2 (28.6)	5 (71.4)
College graduate	28 (100.0)	13 (46.4)	15 (53.6)
Graduate school or higher	9 (100.0)	2 (22.2)	7 (77.8)

## Data Availability

The data can be obtained from the authors upon request.

## Appendix A

The following is a questionnaire about food literacy. In this questionnaire, there are no right or wrong answers. To what extent do you agree or disagree with the following questions?

**Table A1:** Food Literacy Questionnaire.

Food Literacy Question	Strongly Disagree	Disagree	Neutral	Agree	Strongly Agree
	1---------2---------3---------4--------5				
Q1. I know about the various food groups that make up nutritionally balanced meals.					
Q2. I try to eat a variety of food groups such as cereals, fish, vegetables, fruits, and dairy.					
Q3. I make a list of items I need to buy before grocery shopping.					
Q4. I can understand the food labeling in packages of processed foods.					
Q5. When purchasing processed food, I check food information (ingredients, nutrition facts).					
Q6. I check the country of origin when purchasing food.					
Q7. I know how to separate and store ingredients that I cannot consume immediately.					
Q8. I can follow a simple recipe.					
Q9. I can prepare a meal without difficulty.					
Q10. I wash my hands thoroughly before cooking.					
Q11. I know how to store food in refrigerator, or room temperature that can affect freshness and safety.					
Q12. I check the cleanliness of restaurants when eating out.					
Q13. I can understand information related to food safety issues in the media.					
Q14. I can judge critically about the food advertisement content, especially the health claims.					
Q15. Cooking is enjoyable.					
Q16. When eating, I fully concentrate on eating.					
Q17. When eating, I savor various senses such as visual beauty, aroma, taste, and texture.					
Q18. I am grateful for the process that has allowed the food to come to the table.					
Q19. I like to eat or share food with my family, friends, and neighbors.					
Q20. I enjoy talking about food with people around me.					
Q21. I am interested in food from various cultures.					
Q22. Enjoying traditional food can help protect our cultural identity.					
Q23. I know why choosing seasonal food is good for the environment.					
Q24. I think food that is directly traded with producers is more reliable.					
Q25. I think choosing organic products is important for environmental conservation.					
Q26. I think rural farmers are important for a sustainable society.					
Q27. I am interested in urban agriculture (such as city gardening, weekend farming, etc.).					
Q28. It is important to consider animal welfare when purchasing meat and eggs.					
Q29. I know why it is better to choose fair-trade products.					
Q30. I believe that reducing meat and promoting vegetarianism helps slow climate change.					
Q31. I try to reduce food waste.					
Q32. I try to reduce food packaging waste (take-out drinks, delivery foods, etc.).					
Q33. I think everyone should have access to quality food regardless of economic circumstances.					
